# Prostaglandin E2 (PGE2) promotes proliferation and invasion by enhancing SUMO-1 activity via EP4 receptor in endometrial cancer

**DOI:** 10.1007/s13277-016-5087-x

**Published:** 2016-05-26

**Authors:** Jieqi Ke, Yixia Yang, Qi Che, Feizhou Jiang, Huihui Wang, Zheng Chen, Minjiao Zhu, Huan Tong, Huilin Zhang, Xiaofang Yan, Xiaojun Wang, Fangyuan Wang, Yuan Liu, Chenyun Dai, Xiaoping Wan

**Affiliations:** 1 Department of Obstetrics and Gynecology, Shanghai General Hospital, Shanghai Jiao Tong University School of Medicine, Shanghai, China; 2 Department of Obstetrics and Gynecology, Shanghai First Maternity and Infant Hospital affiliated with Tong Ji University, No. 536, Changle Road, Jing’an District, Shanghai, China

**Keywords:** Prostaglandin E2, SUMO-1, Endometrial cancer, EP4, β-catenin

## Abstract

Prostaglandin E2 (PGE2), a derivative of arachidonic acid, has been identified as a tumorigenic factor in many cancers in recent studies. Prostaglandin E synthase 2 (PTGES2) is an enzyme that in humans is encoded by the *PTGES2* gene located on chromosome 9, and it synthesizes PGE2 in human cells. In our study, we selected 119 samples from endometrial cancer patients, with 50 normal endometrium tissue samples as controls, in which we examined the expression of PTGES2. Both immunohistochemistry (IHC) and Western blot analyses demonstrated that synthase PTGES2, which is required for PGE2 synthesis, was highly expressed in endometrium cancer tissues compared with normal endometrium. Stable PTGES2-shRNA transfectants were generated in Ishikawa and Hec-1B endometrial cancer cell lines, and transfection efficiencies were confirmed by RT-PCR and Western blot analyses. We found that PGE2 promoted proliferation and invasion of cells in Ishikawa and Hec-1B cells by cell counting kit-8 tests (CCK8) and transwell assays, respectively. PGE2 stimulation enhanced the expression of SUMO-1, *via* PGE2 receptor subtype 4 (EP4). Further analysis implicated the Wnt/β-catenin signaling pathway function as the major mediator of EP4 and SUMO-1. The increase in SUMO-1 activity prompted the SUMOlyation of target proteins which may be involved in proliferation and invasion. These findings suggest SUMO-1 and EP4 as two potential targets for new therapeutic or prevention strategies for endometrial cancers.

## Introduction

Endometrial cancer is the most common tumor of the female reproductive system in developed countries [1]. In the US, endometrial cancer results in about 8,590 death cases per year [2], while in developing countries, such as China, the mortality incidence is approximately 7.44/10^5^ people [3].

Prostaglandin E2 (PGE2) is the most abundant prostanoid in the human body and exhibits the most versatile functions ranging from reproduction to neuronal, metabolic, and immune functions [4]. Secreted PGE2 acts in either an autocrine or paracrine manner through its four cognate G protein-coupled receptors, EP1 to EP4. Many studies have found PGE2 associated with tumors of the colorectal organs, lung, and breast [5–7]. In some before studies of our research teams, we have found some cytokines high expressed in endometrial cancers [8, 9], and PGE2 is one of them. Some previous studies have also suggested that PGE2 participates in tumorigenesis of endometrial cancers [10, 11]; however, the definite effect and its detailed mechanisms are unclear, which promotes us interest in PGE2.

Dynamic chromatin structure regulation by post-translational protein modifications (PTPM) modulates the accessibility of DNA and consequently the transcription of genes. Small ubiquitin-like modifier (SUMO) modification in the epigenetic regulation of chromatin states has been extensively studied [12]. SUMOlyation of specific transcription factors or chromatin remodeling proteins, in most cases, is associated with repressive complex formation and a silencing role in transcription regulation [13, 14]. In humans, three main subtypes of SUMOs have been identified: SUMO-1, SUMO-2, and SUMO-3 [15]. SUMO-1 is a highly conserved modifier that can covalently conjugate to a variety of cellular proteins [16–18]. One obvious function of SUMO-1 is its capability to modify p53 and enhance transcriptional activity [19]. As p53 is often mutated in endometrial cancer, the likelihood that SUMO-1 has an important role in endometrial cancers is high.

Herein, we examined the expression and effect of PGE2 on endometrial cancer cells. This study investigated the role of PGE2, via its receptor EP4, in the promotion of SUMO-1 expression, and identified that this regulation occurs through Wnt/β-catenin signaling pathway, resulting in the enhancement of proliferation and invasion of endometrial cancer cells.

## Materials and methods

### Reagents and antibodies

Prostaglandin E2 was from Sigma (St. Louis, MO). Sulprostone (Sulp) was from ABCAM (Cambridge, UK). Butaprost (Buta) was from Santa Cruz Biotechnology (Dallas, USA). Prostaglandin E1 Alcohol (POH), L161982, AZD5363, FH535, and ICI 182780 (ICI) were from Cayman Chemical (Detroit, USA). Antibody of prostaglandin E synthase 2 (Anti-PTGES2) was from Proteintech (Chicago, USA). Antibodies of prostaglandin E receptor 4 (Anti-EP4), SUMO-1, and SUMO-2, 3 were from ABCAM (Cambridge, UK). ELISA Kit for Prostaglandin E2 (PGE2) was from Cloud-Clone Corp (Houston, USA).

### Patients and samples

Tissues samples for immunohistochemistry (IHC) and western blot were obtained from 119 patients with endometrial cancer and 50 patients with normal endometrium who underwent surgical resection at Shanghai General Hospital from 2005 to 2014. The project was approved by the Human Investigation Ethics Committee of the Shanghai General Hospital, and informed consent was obtained from all patients before the study.

### Cell lines and culture conditions

The human endometrial Ishikawa cell lines were obtained from Dr. Qi Che (Shanghai Jiao Tong University, Shanghai, China). Ishikawa cells were grown in DMEM/F12 (Gibco, Auckland, NZ) supplemented with 10 % fetal bovine serum (FBS) (Gibco, Carlsbad, CA). Cells were incubated at 37 °C in a humidified atmosphere containing 5 % CO_2_. All experiments were performed at the third passage after thawing.

### Total RNA extraction, real-time RT-PCR

Total RNA from Ishikawa cells was isolated by Trizol (15596-026, Invitrogen) and cDNA as prepared using the reverse transcriptase kit. Real-time reverse transcription (RT)-PCR was conducted using an ABI Prism 7500 sequence detection system (Applied Biosystems, Foster City, CA) and performed with SYBR Green PCR Master Mix (Toyobo, Osaka, Japan). A comparative CT method was used to analyze the relative changes in gene expression. The results were expressed relative to the number of GAPDH transcripts (internal control). Sequences of the primer pairs used are listed in Table 1.

**Table 1:** Primer sequences for real-time PCR analysis

	Forward (5′–3′)	Reverse (5′–3′)
PTGES2	CTTCCTTTTCCTGGGCTTCG	GAAGACCAGGAAGTGCATCCA
GAPDH	GAAGGTGAAGGTCGGAGTC	GAAGATGGTGATGGGATTTC
shPTGES2	GCAAUAAGUACUGGCUCAUTT	AUGAGCCAGUACUUAUUGCTT
SUMO-1	ACCGTCATCATGTCTGACCA	TGGAACACCCTGTCTTTGAC
SUMO-2	TCCCCGCGCCGCTCGGAATCCATGTCCGAG	CCCGAATTCGGGACGGGCCCTCTAGAAACT
SUMO-3	GAGGAGACTCCGGCGGGATCCATGGCCGACGAA	GTAGAATTCCAGGTTCCCTTTTCAGTAGAC
siSUMO-1	UCAAGAAACUCAAGAAUC	UUCUCCGAACUUGUCACAUUU

### Western blot

For Western blot analysis, cells were lysed in lysis buffer for 30 min at 4 °C. Total proteins were fractionated by SDS–PAGE and transferred onto PVDF membrane. The membranes were then incubated with appropriate primary antibodies (PTGES2, EP4, and GAPDH), followed by incubation with horse-radish peroxidase-conjugated secondary antibody (Santa Cruz Biotechnology). The probed proteins were detected by enhanced chemiluminescent reagents. GAPDH was used as an internal control.

### Immunohistochemistry (IHC)

Staining was performed on paraffin-embedded specimens using primary antibodies as follows: anti-PTGES2 (1:100; Proteintech). The percentage of positively stained cells was rated as follows: 0 point = 0 %, 1 point = 1 % to 25 %, 2 points = 26 % to 50 %, 3 points = 51 % to 75 %, and 4 points = greater than 75 %. The staining intensity was rated in the following manner: 0 points = negative staining, 1 point = weak intensity, 2 points = moderate intensity, and 3 points = strong intensity. Then, immunoreactivity scores for each case were obtained by multiplying the values of the two parameters described above. The average score for all of five random fields at ×200 magnification was used as the histological score (HS) as before researches [8]. Tumors were categorized into two groups based on the HS: low-expression group (HS < 6) and high-expression group (HS ≥ 6). The results of IHC were analysed with chi-square test.

### Cell proliferation

The cell proliferation was examined with the CCK-8 (Cell Counting Kit-8) assay (Dojindo, Kumamoto, Japan) according to the manufacturer’s protocol. After 24, 48, 72, or 96 h of incubation, the supernatant of each group was removed, and cells were incubated in DMEM medium containing CCK-8 for another 2 h at 37 °C. The optical density (OD) value for each well was read at 450 nm using an automated microplate reader (Sunrise, Tecan, Switzerland).

### Transwell invasion assays

For transwell invasion assays, the upper side of an 8-μm pore, 6.5-mm polycarbonate transwell filter (Corning, New York, NY) chamber was uniformly coated with Matrigel basement membrane matrix (BD Biosciences, Bedford, MA) for 2 h at 37 °C before cells were added. A total of 2 × 10^4^ cells were seeded into the top chamber of a transwell filter (in triplicate) and incubated for 48 h. Invasive cells, which were on the lower side of the filter, were fixed in 4 % paraformaldehyde, stained in 0.5 % crystal violet (Beyotime), and counted using a microscope. A total of five fields were counted for each transwell filter. Each field was counted and photographed at ×200 magnification.

### Transfection

To inhibit the expression of target gene, we designed and prepared HPLC-purified siRNAs according to the sequence of the target gene. A scrambled siRNA with no homology to any known human mRNA was used as negative control. siRNA oligonucleotide duplexes were synthesized by GenePharma Biotech (Shanghai, China). The sequences of siRNA oligos are provided in Table 2. Cells were seeded in 6-well plates at 70–80 % confluence and grown overnight before transfection. Transfection of cells with the siRNA or non-target control (siCo) was accomplished using the lipfectamine 2000 transfection reagent (Invitrogen, Carlsbad, CA, USA) according to the manufacturer’s instructions.

**Table 2:** Expression of PTGES2 in normal endometrium and endometrial cancer

Groups	Patients	Histological score (HS) of PTGES2		*χ* ^2^
		low group (HS < 6)	High group (HS ≥ 6)	*P*
Normal endometrium	50	36	14	
Endometrial cancer	119	46	73	<0.001

### Enzyme-linked immunosorbent assay (ELISA)

PGE2 levels were detected in culture medium using solid phase sandwich enzyme-linked immunosorbent assay (ELISA) assays according to the manufacturer’s protocol (Cloud-Clone Corp). The PGE2 assay sensitivity was 0.1 pg/ml, and the assay range was 1.03–4000 pg/ml. For the statistical analysis, culture medium was collected three times independently.

### Statistical analyses

Continuous variables were recorded as mean ± SD and analyzed with the Student’s *t* test. Data was analyzed by unpaired Student’s *t* test or by one-way analysis of variance (ANOVA). The *χ* ^2^ test for tables was used to compare the categorical data. All statistical analyses were done using Statistical Package for the Social Sciences version 17.0 (SPSS, Chicago, IL). The *P* values < 0.05 were considered statistically significant. All experiments were performed at least three times.

## Results

### PTGES2 is highly expressed in human endometrium cancer tissues and cell lines

**Fig. 1:**
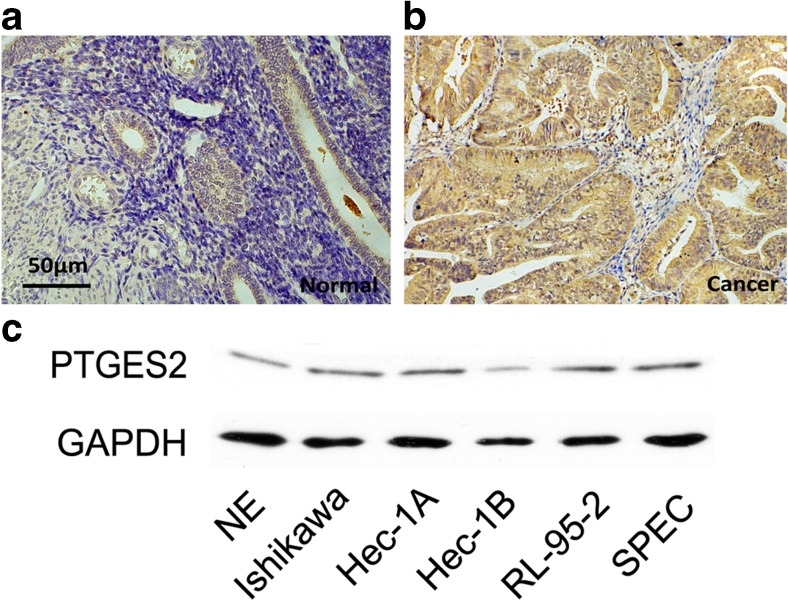
PTGES2 expressions in normal endometrium and endometrial cancer tissues **a** and **b** Immunohistochemistry tests for normal endometrium from curettage patients and cancer tissues from endometrial cancer patients. **c** Western blot tests for PTGES2 expression in Ishikawa, Hec-1A, Hec-1B, RL-95-2, and SPEC cells

Prostaglandin E synthase 2 (PTGES2) is involved in the synthesis of PGE2. Recent studies have suggested that PGE2 may be a mitogen associated with a variety of tumors [5–7, 20]. We performed immunohistochemistry (IHC) in normal endometrium and endometrial cancer tissues. Compared with the normal endometrium, the expression of PTGES2 was significantly upregulated in the endometrial cancer tissues (Fig. 1a, b; Table 2).By statistical analysis, we found that increased expression of PTGES2 was notably associated with the tumor stage (*P* = 0.0088), grade (*P* = 0.0104), and the depth of myometrial invasion (*P* = 0.0015), but not with other characteristics (Table 3). We also examined the expression of PTGES2 in several human endometrial cancer cell lines (Fig 1c), with protein from normal endometrium (NE) as control. PTGES2 expression was high in these human endometrial cancer cell lines, with the highest levels in Ishikawa cells. And we chose Ishikawa cells in following research.

**Table 3:** Relationships between PTGES2 expression and clinicopathologic characteristics in endometrial cancer

Characteristics	Patients	Histological score (HS) of PTGES2		*χ* ^2^
		low group (HS < 6)	High group (HS ≥ 6)	*P*
Total	119	46	73	
Age (years)				
≥55	66	21	45	0.0876
<55	53	25	28	
FIGO stage				
Stage I–II	73	35	38	0.0088
Stage III–IV	46	11	35	
Grade				
G1–G2	82	38	44	0.0104
G3	37	8	29	
Myometrial invasion				
≤1/2	69	35	34	0.0015
>1/2	50	11	39	
Nodal metastasis				
Positive	21	9	12	0.6639
Negative	98	37	61	

### Prostaglandin E2 increases proliferation and invasion potential of human endometrial cancer cells

**Fig. 2:**
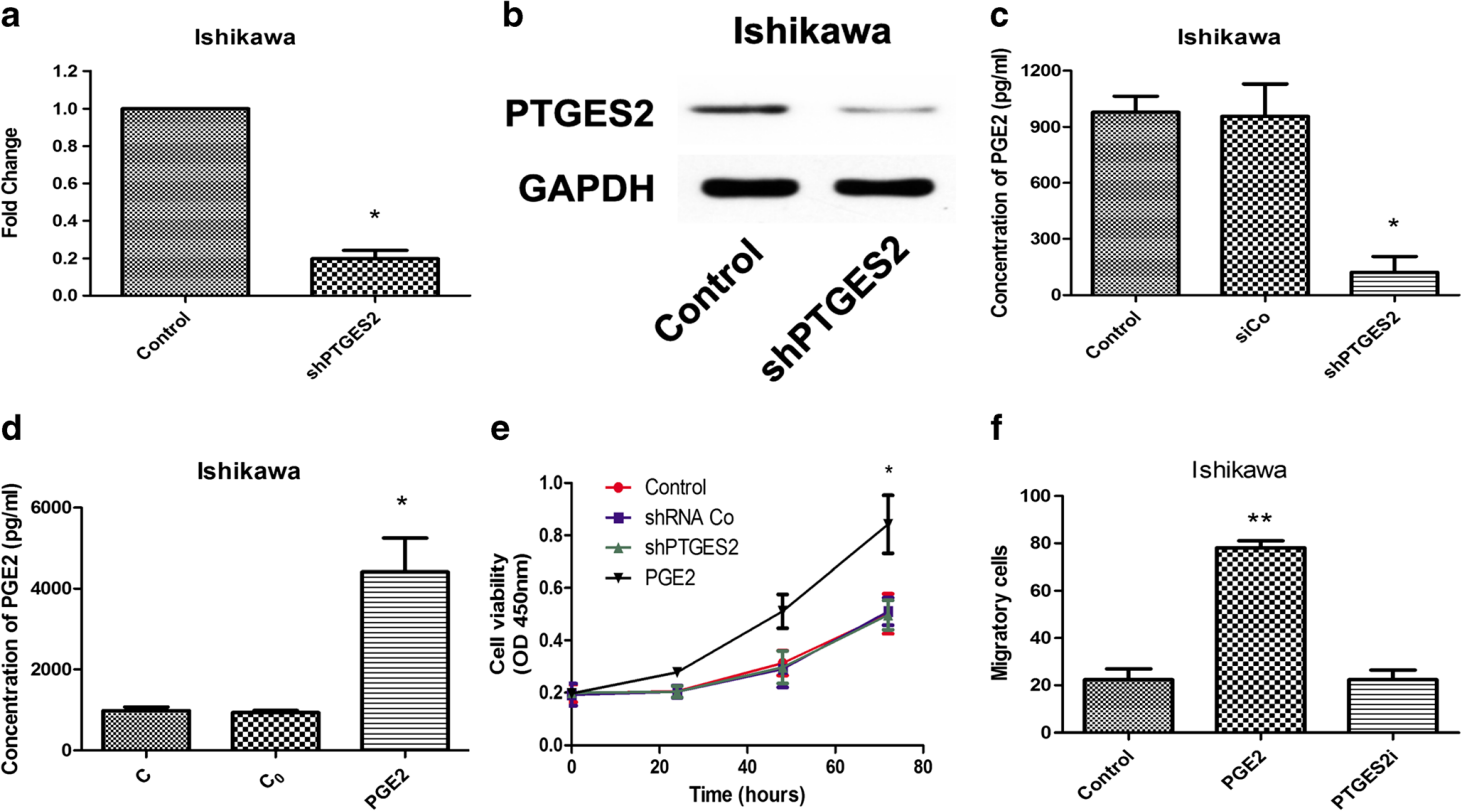
Prostaglandin E2 raises proliferation and invasion in human endometrial cancer cells **a** RT-PCR analysis for Ishikawa cells after transfection of PTGES2 shRNAs. *p < 0.05 versus control group, tested with unpaired Student’s *t* test. **b** Western blot tests for Ishikawa cells after transfection of PTGES2 shRNAs. **c** ELISA for PGE2 concentration in shRNAs transfected Ishikawa cells after 24 h of culture. *p < 0.05, analyzed by one-way analysis of variance (ANOVA). **d** ELISA for PGE2 concentration in PGE2 stimulated Ishikawa cells after 24 h of culture. C replicated control group, in which no PGE2 stimulated. C_0_ replicated PGE2 working concentration. *p < 0.05, analyzed by one-way analysis of variance (ANOVA). **e** CCK8 assays were conducted at each time point to quantify cell viability for Ishikawa cells transfected with control or PTGES2 shRNA, or stimulated with PGE2. *p < 0.05, analyzed by one-way analysis of variance (ANOVA). **f** Transwell for Ishikawa cells with PGE2 stimulated or transfected with PTGES2 shRNA. Figure shows the number of invasive cells for each group (averaged across five random images). **p < 0.01, analyzed by one-way analysis of variance (ANOVA)

We initially selected human endometrial cancer cells in which we performed stable depletion of PTGES2 for subsequent experiments. To achieve this, we designed three different pairs shRNAs, and choose the most effective shRNA. As shown in the representative RT-PCR (Fig 2a) and Western blot (Fig 2b), PTGES2 expression was knocked down by shRNA transfection. We also examined the expressed of PGE2 in PTGES2-shRNA transfected Ishikawa cells (Fig 2c). As expected, PGE2 concentration decreased in shPTGE2-Ishikawa cells, which further confirmed that PTGES2 controls PGE2 synthesis in the cell lines. What is more, we examined PGE2 concentration in the control group, C_0_ group, and PGE2-stimulated group (Fig 2d). C_0_ group represent the PGE2 stimulating concentration in culture without cells. Here we can see that, PGE2 concentration in PGE2 stimulated group was much higher than control group and C_0_ group, which promotes a high concentration of PGE2 can be created with adding exogenous PGE2 while shPTGES2 creates low PGE2 concentration. We then assessed the proliferation and invasion of endometrial cancer cells with or without PGE2 stimulation. A significant increase in proliferation of Ishikawa cells was observed under PGE2 stimulation (1 × 10^−9^ mol/L), which was abolished in shPTGE2-Ishikawa cells (Fig 2e). The PGE2 concentrations used here were similar as in previously reported studies [21, 22]. Additionally, PGE2 also increased the invasion of Ishikawa cells (Fig. 2f), observations that were significantly inhibited in shPTGE2 Ishikawa cells (Fig 2f).

### PGE2 promotes proliferation and invasion via EP4

**Fig. 3:**
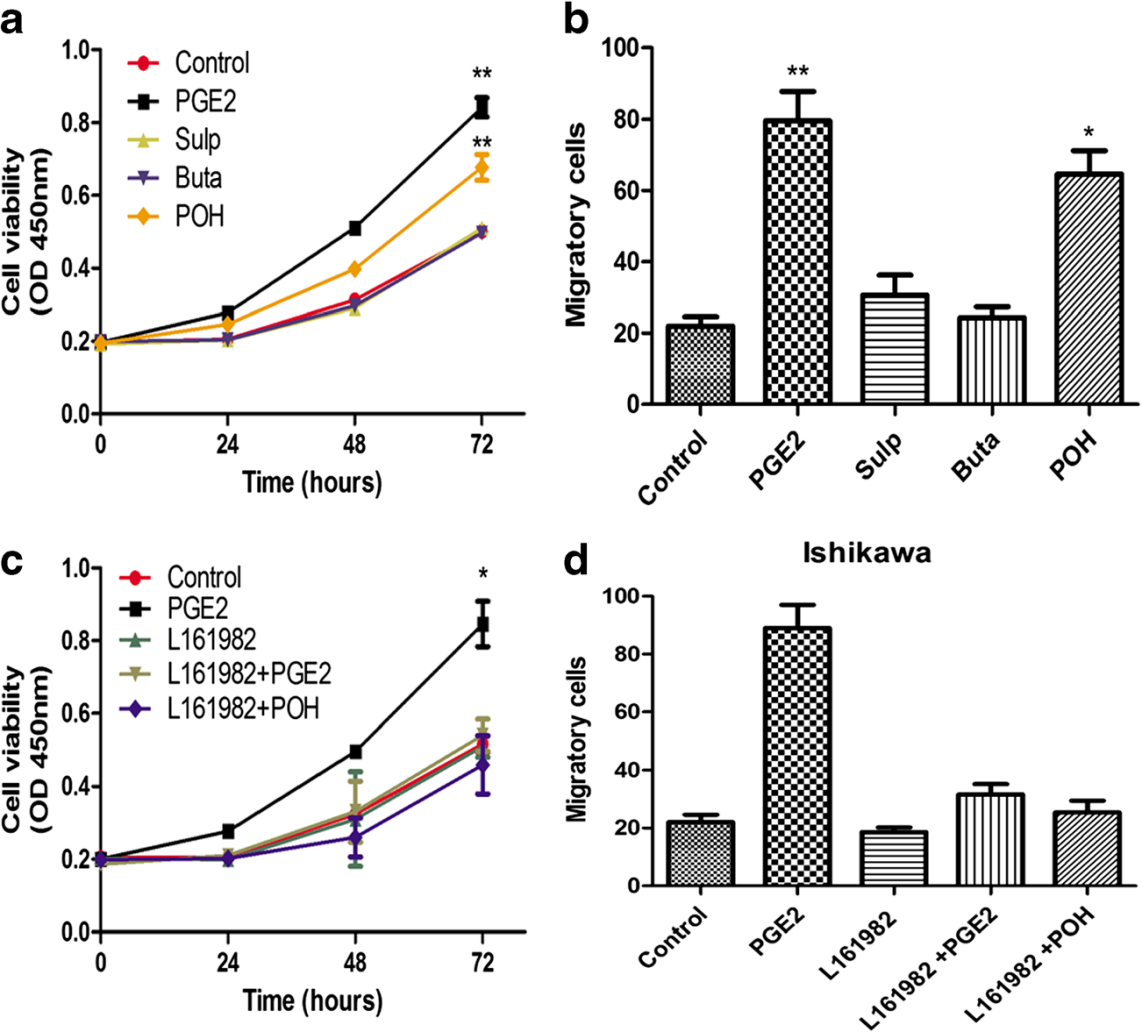
PGE2 raises proliferation and invasion via EP4 **a** CCK8 assays were conducted at each time point to quantify cell viability for Ishikawa cells transfected after clutching with three EP agonists. **p < 0.01, analyzed by one-way analysis of variance (ANOVA). **b** Transwell for Ishikawa cells after clutching with three EP agonists. Figure shows the number of invasive cells for each group (averaged across five random images). *p < 0.05, **p < 0.01, analyzed by one-way analysis of variance (ANOVA). **c** CCK8 assays were conducted at each time point to quantify cell viability for Ishikawa cells transfected after treated with EP4 antagonist L161982, together with or without PGE2 and POH. *p < 0.05, analyzed by one-way analysis of variance (ANOVA). **d** Transwell for Ishikawa cells after adding EP4 antagonist L161982, treated with or without PGE2 and POH. Figure shows the number of invasive cells for each group (averaged across five random images)

As there are four different subtypes of the PGE2 receptor, EP1 to EP4, we used three selective EP agonists to determine the most effective EP. Of the three agonists, sulprostone (Sulp) bound EP1 and EP3, butaprost (Buta) bound EP2, while prostaglandin E1 alcohol (POH) ligated EP4. Fig. 3 shows proliferation and invasion of Ishikawa cells after stimulation with the three different EP agonists (1 × 10^−9^ mol/L). The proliferation of cells increased in response to POH and was at similar levels to PGE2, while cells in sulp- and buta-stimulated groups did not exhibit significant differences in comparison to control groups (Fig. 3a). We also observed a similar trend in the invasion assay, wherein a comparable rate between PGE2 and POH was seen (Fig. 3b). Subsequent experiments focused on EP4, and L161982 was chosen as an EP4 antagonist (10 × 10^−9^ mol/L), for rescue experiments. The increased proliferation and invasion rates from PGE2 stimulation were lost after L161982 treatment (Fig. 3c, d). As the result, EP4 was considered to be the modulating EP in the PGE2-induced effect on proliferation and invasion of human endometrial cancer cells.

### PGE2 promotes endometrial cancer cell proliferation and invasion by stimulating SUMO-1

**Fig. 4:**
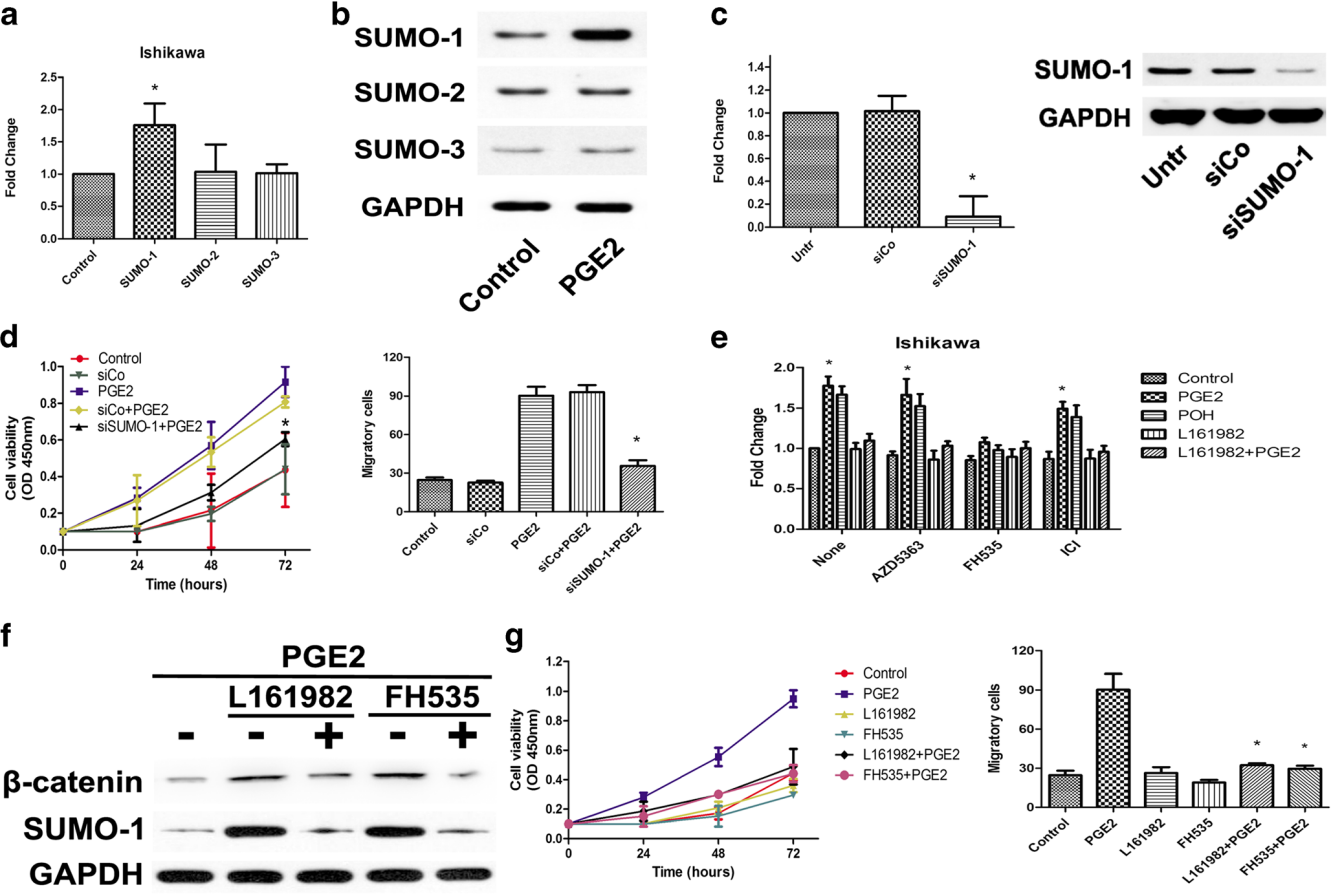
PGE2 promotes endometrial cancer cell proliferation and invasion by stimulates SUMO-1 **a** RT-PCR analysis for three SUMO subtypes in Ishikawa cells after PGE2 stimulated. *p < 0.05, analyzed by one-way analysis of variance (ANOVA). **b** Western blot tests for three SUMO subtypes in Ishikawa cells after PGE2 stimulated. **c** RT-PCR analysis and western blot tests in Ishikawa cells after transfected with SUMO-1 siRNAs. *p < 0.05 versus control group, tested with unpaired Student’s *t* test. **d** CCK8 assays were conducted at each time point to quantify cell viability for Ishikawa cells after transfection of SUMO-1 siRNAs. *p < 0.05, analyzed by one-way analysis of variance (ANOVA). **e** Ishikawa cells were treated with AZD5363 (1 × 10^–6^ mol/L), FH535 (1.5 × 10^–6^ mol/L), and ICI (1 × 10^−6^ mol/L) for 24 h. Then stimulated with PGE2, POH, or L161982. RT-PCR analysis for SUMO-1 in Ishikawa cells. *p < 0.05, analyzed by one-way analysis of variance (ANOVA). **f** Ishikawa cells were treated with FH535 (1.5 × 10^–6^ mol/L) or L161982 (10 × 10^–9^ mol/L) for 24 h, then stimulated with or without PGE2. Western blot tests for β-catenin and SUMO-1 in Ishikawa cells. **g** CCK8 assays were conducted at each time point to quantify cell viability for Ishikawa cells treated with PGE2 (1 × 10^–9^ mol/L), FH535 (1.5 × 10^–6^ mol/L), or L161982 (10 × 10^–9^ mol/L). Transwell for Ishikawa cells treated with PGE2 (1 × 10^–9^ mol/L), FH535 (1.5 × 10^–6^ mol/L) or L161982 (10 × 10^−9^ mol/L). Figure shows the number of invasive cells for each group (averaged across five random images). *p < 0.05, analyzed by one-way analysis of variance (ANOVA)

To determine the downstream target of EP4, we analyzed the SUMOs in PGE2-stimulated Ishikawa cells. Some before researches had showed SUMOs act an important role in oncogenesis and human endometrium proliferation [23–25], and SUMOylation had potential connect with COX and PGE2 in gynecology tumors such as ovarian cancers [26]. The mRNA of SUMO-1 was increased in PGE2-stimulated group while SUMO-2 and SUMO-3 showed no obvious changes (Fig 4a), results that were confirmed by Western blot analysis (Fig 4b). To further examine the role of SUMO-1 in PGE2-induced proliferation and invasion, three SUMO-1 siRNA primer pairs were designed and the most effective siRNA was used in further experiments, whose transfection efficiency was confirmed by mRNA and protein level analyses (Fig 4c). The results of CCK-8 and transwell assays showed that SUMO-1 could promote proliferation and invasion in endometrial cancer cells (Fig 4d). To further assess the signaling pathway involved in the interaction of EP4 and SUMO-1, Ishikawa cells were treated with three different signal inhibitors, AZD5363 (Akt inhibitor, 1 × 10^−6^ mol/L), FH535 (β-catenin inhibitor, 1.5 × 10^−6^ mol/L) and ICI (estrogen receptor antagonist, 1 × 10^−6^ mol/L), and we examined the gene expression of SUMO-1 (Fig 4e). The results showed that SUMO-1 mRNA expression was inhibited by FH535 treatment, which suggested that the Wnt/β-catenin signaling pathway was the mediator for EP4 and SUMO-1. We further examined SUMO-1 protein levels with Western blot (Fig 4f) to confirm the role of Wnt/β-catenin signaling pathway in cells. Finally, the results of CCK-8 and transwell analyses showed that SUMO-1 can promote proliferation and invasion in endometrial cancer cells through Wnt/β-catenin signaling pathway (Fig 4g).

## Discussion

The mediators and cellular effectors of inflammation are important constituents of the local tumor microenvironment [27]. Tumor-promoting inflammation is an accepted enabling characteristic of cancers [28]. Some recent studies have shown the relationship between cancers and inflammatory factors, such as IL-6 [8] and IL-10 [29]. As a classical cytokine, prostaglandin was first identified in 1936 [30]. A series of studies of prostaglandin in cancers showed that the subtype of EPs effect in different cancers was variable [5, 6, 31]. A recent study showed that PGE2 induces lung metastasis in breast cancers via EP2 [32]. However, in endometrial cancers, no result of PGE2 functional activation has ever been reported. Our work uncovered that PGE2 promotes the proliferation and invasion of endometrial cancer cells via its receptor subtype, EP4. Based on our results, we examined two points in the mechanism: EP4 and SUMO-1. In this mechanism, EP4 act as a gate of the effect while high PGE2 concentration of tumor microenvironment influences the outcome of endometrial cancer cells.

In our study, first we showed that PTGES2 is highly expressed in endometrial cancer both in tissue and in cell lines. Further experiments uncovered EP4 as the key receptor in the PGE2 effect which influenced endometrial cancer cells proliferation and invasion. In previous studies, PGE2 was thought to play an important role in most gynecological tumors via different receptors [7, 33]. However, EP2 does not effect these changes, which was not the same as some previous studies in many other cancers [10, 11, 34]. This suggested that a new therapeutic opportunity might exist for patients with endometrial cancers and prevention for those in high-risk groups. In 2005, Francesmary Modugno et al. recommended that inflammation may work in conjunction with, or in addition to, estrogen exposure in the development of endometrial cancer [35]. Some recent clinical researches also showed that the use of aspirin, but not non-aspirin NSAIDs, may reduce the risk of endometrial cancer [36]. However, as is known, NSAIDs increase gastrointestinal discomfort and may aggravate illness in ulcer patients. EP4 may therefore be a new target for further treatment with fewer side effects in both treatment and prevention.

Another key point of the mechanism in our study was SUMO-1, whose expression was promoted by PGE2, and further enhanced proliferation and invasion. As is known, SUMOs contribute to protein modification, especially in transcription, chromosome organization, and DNA repair [37]. A recent study suggested that SUMO-1 promotes cancer cells proliferation and apoptosis by increasing SUMOylation of histone H4 [38]. This research demonstrated the importance of histone modification, which conclusion motivated our study. In 2012, Picard et al. determined that estrogen receptor β was a SUMOylation target of SUMO-1[39]. We have known the relationship between estrogen and endometrial cancer and their consequence points to SUMO-1, and likely integrates several exogenous cytokine signals. A study from Oxford showed that chromatin modification by SUMO-1 stimulates the promoters of translation machinery genes [40]. In addition to gene expression, the changes of cancer cell metabolism in hypoxia environment are also related to SUMO-1 function of [41]. All these studies demonstrated that SUMO-1 is an important point in oncogenesis, and the downstream targets of SUMO-1 are flexible and worth further studies.

We discovered that Wnt/β catenin signaling pathway acts an important role between EP4 and SUMO-1. As is known, the Wnt signaling pathway is closely related with many human diseases such as colorectal cancer [42] and glioblastoma [43]. Wnt signaling has also been examined in endometrial cancers. Some recent studies had reported that Wnt pathway inhibitors expression was down-regulated in cancer patients [44]. We find β-catenin expression reduced after inhibition of EP4, and SUMO-1 expression reduced upon inhibition of β-catenin. However, Wnt/β-catenin signaling pathway may not be the only signal pathway in EP4 regulation to SUMO-1. In our study, while we examined three major signal pathways, this is not exhaustive and we therefore cannot rule out other unexamined pathways that may contribute to EP4 regulation to SUMO-1. Future studies should explore these additional pathways.

In summary, we demonstrated a likely mechanism of proliferation and invasion in endometrial cancer cells. PGE2 promoted proliferation and invasion by enhancing SUMO-1 via EP4 receptor in endometrial cancer. The main modulators, EP4 receptor and SUMO-1, may be regulated by Wnt/β-catenin signaling pathway, and are potentially two new targets for treatment and prevention of endometrial cancers. These findings also suggest that inflammation in tumor microenvironment and protein modifications considerably contribute to tumorigenesis and development.
